# Longitudinal time course of muscle impairments during partial weight-bearing in rats

**DOI:** 10.1038/s41526-019-0080-5

**Published:** 2019-08-22

**Authors:** Marie Mortreux, Frank C. Ko, Daniela Riveros, Mary L. Bouxsein, Seward B. Rutkove

**Affiliations:** 1 Department of Neurology, Harvard Medical School – Beth Israel Deaconess Medical Center, Boston, MA USA; 2 Department of Orthopedics, Center for Advanced Orthopaedic Studies, Harvard Medical School – Beth Israel Deaconess Medical Center, Boston, MA USA

**Keywords:** Experimental models of disease, Physiology

## Abstract

In the near future, space agencies plan to send the first crews for extended stays on the Moon and Mars, where gravity is significantly reduced compared to Earth (0.16×*g* and 0.38×*g*, respectively). However, the long-term effects of partial gravity have not yet been elucidated, and ensuring astronauts’ health and performance is crucial to the success of these missions. Using a quadrupedal partial weight-bearing (PWB) model in rats that we designed, we investigated the longitudinal time course of muscle function at three different PWB levels. We demonstrated that both muscle mass and muscle function are significantly impaired in reduced weight-bearing environments as early as after 7 days of suspension. Moreover, we showed that muscular alterations are correlated to the PWB level and do not reach a plateau during a 1-month exposure to reduced weight-bearing, emphasizing the need for mitigating countermeasures for safe and successful extraterrestrial exploration.

## Introduction

By providing a sustained mechanical load, gravity is crucial for the maintenance and health of the musculoskeletal system. Microgravity, accordingly, markedly alters musculoskeletal physiology.^1–3^ For example, the soleus mass decreases by over 30% after 3 weeks in microgravity,^4^ and femoral bone strength declines by ~2% for every 100 days in space.^5,6^ But the most important consequences lie in the risks encountered when astronauts return to Earth and are subjected to standard gravity; indeed, the dramatic decrease in muscle size, strength, and endurance^7^ produces long-term consequences including chronic muscle fatigue and persistent low back pain.^8,9^

While considerable information has been gathered concerning the physiological adaptations of the human body to weightlessness,^10,11^ long-term studies are rare and longitudinal assessments remain limited due to the difficulty to obtain muscle biopsy data on astronauts in-flight. For this reason, researchers have relied on several animal models, most notably the rat hindlimb unloading (HLU) protocol developed in 1979 by Morey et al. which has been established as the standard approach^12,13^ for ground-based research. However, this model puts considerable stress on the rat tail, which can lead to blood flow restriction, inflammation, and necrosis. More importantly, the HLU model is not suitable to investigate the effects of partial gravity that will be encountered when astronauts reach extra-terrestrial targets such as Mars (38% of Earth gravity, or 0.38×*g*) and the Moon (16% of Earth gravity, or 0.16×*g*), or when they are exposed to artificial gravity induced by continuous centrifugation.^14^ An innovative partial weight-bearing (PWB) model, incorporating a tail-suspension and body harness, has been developed in mice, allowing normal activity for up to 3 weeks at several different levels of PWB.^15,16^ However, rats are generally considered a better animal model for spaceflight studies since their physiological processes, including drug metabolism, are considered more analogous to those of humans.^17^ Accordingly, we recently modified this mouse PWB model for rats^18^ by substituting a pelvic harness for the tail suspension, thereby providing a less stressful experience with improved reliability.

In the present study, we sought to extend our initial^18^ work for up to 4 weeks and assessed the functional muscle alterations that occur in three different PWB levels (70%, 40%, and 20% of normal loading). Additionally, we harvested muscle tissues on days 7, 14, and 28 to obtain a comprehensive timeline of muscle atrophy at these different levels of PWB. By doing so, we can provide a detailed picture of the muscle alterations induced by a reduction of the mechanical load. This longitudinal study provides the first step for better understanding the impact of space missions in which stays on the Moon and Mars are planned.

## Results

**Fig. 1:**
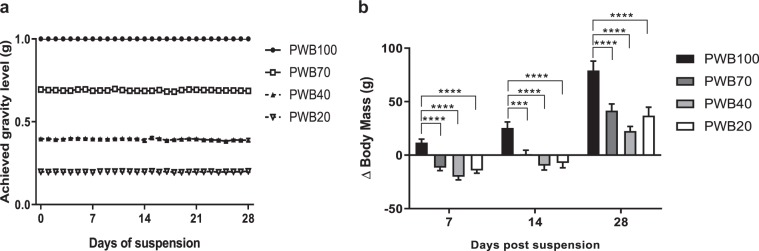
Tolerability and stability of the PWB model over time. Daily achieved partial weight-bearing (**a**) and weekly change in body mass (**b**) of all the animals involved in the study for up to four weeks (*n* = 11–12 per group). Results are presented as mean ± SEM and Tukey’s post hoc tests following two-way ANOVA are represented as ***: *p* < 0.001, ****: *p* < 0.0001

The consistency of the PWB level achieved was high (Fig. 1a). Indeed, all unloaded groups displayed minimal differences between their achieved and desired PWB level, which was further demonstrated by low coefficients of variation (PWB70: 0.54%, PWB40: 1.20%, PWB20: 0.95%). Actual body mass without the harness was recorded weekly (Fig. 1b), and at all time-points, PWB animals displayed a significantly different body mass change compared to the normal loading (PWB100) controls. Despite exhibiting a transient and significant loss of body mass in the first 14 days, all groups reached a body mass significantly above their baseline but lower than the PWB100 control rats after 28 days of suspension.

**Fig. 2:**
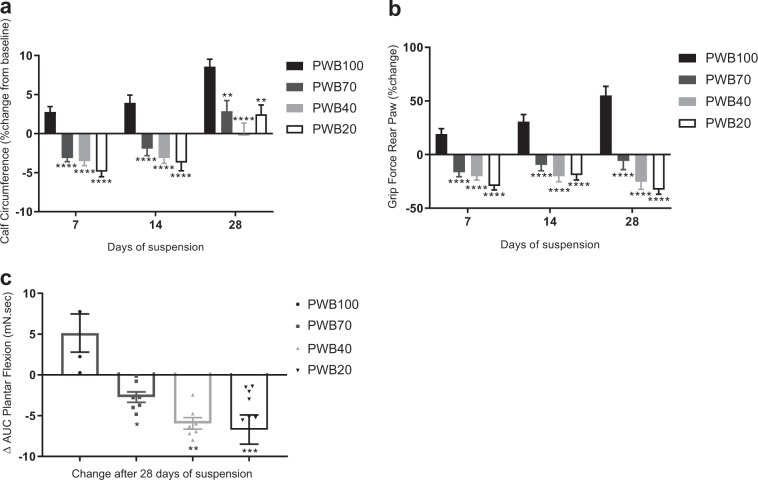
Longitudinal assessment of muscle function. Weekly change in calf circumference (**a**) and rear paw grip force (**b**) of all animals (*n* = 11–12 per group). 4-weeks change in the area under curve (AUC) obtained during the tetanic plantar flexion (**c**, *n* = 3–6 per group). Results are presented as mean ± SEM and Tukey’s post hoc tests following one-way ANOVA (**c**) or two-way ANOVA (**a**, **b**) are represented as *: *p* < 0.05, **: *p* < 0.01, ***: *p* < 0.001, and ****: *p* < 0.0001 vs PWB100

Calf circumference (Fig. 2) steadily increased over time in the control (PWB100) group, while it decreased significantly during the first 2 weeks in all PWB groups compared to their baseline (Fig. 2a). After 28 days of suspension, all animals displayed a calf circumference greater than their baseline, but control group values remained significantly higher than all other values. From 1-week onward, all PWB groups displayed a significantly lower grip force compared both to the controls (PWB100) and their respective baseline values (Fig. 2b).

Nerve-stimulated plantar flexion force was measured only on days 0 and 28 (Fig. 2c). The control animals (PWB100) showed an increase in their area under curve (AUC) after 4 weeks (+5.1 mN s), which was significantly greater than in all the other PWB groups. Each unloaded group displayed a reduction of the AUC in a dose-dependent manner after 28 days of suspension, ranging from −2.7 mN s for the PWB70 groups and −5.9 mN s for the PWB40 group, to −6.7 mN.s for the PWB20 animals. This dose-dependent decrease over a 1-month period was assessed by performing a linear regression among the groups (*R*^2^ = 0.98, *p* < 0.0001). Additionally, the maximal impulse recorded at 28 days of suspension can be normalized by the combined wet mass of the gastrocnemius and soleus muscles (triceps surae) in order to determine muscle quality (Supplementary Table 1). This parameter highlighted the linear correlation (*p* = 0.043 using a one-way ANOVA followed by a post hoc test for trend) between the different groups, ranging from 9.87 ± 1.64 mN s g^−1^ for the control group to 6.14 ± 1.25 mN s g^−1^ for the rats exposed to PWB20, suggesting a reduction in the intrinsic force generating capability of the muscle due to PWB.

**Fig. 3:**
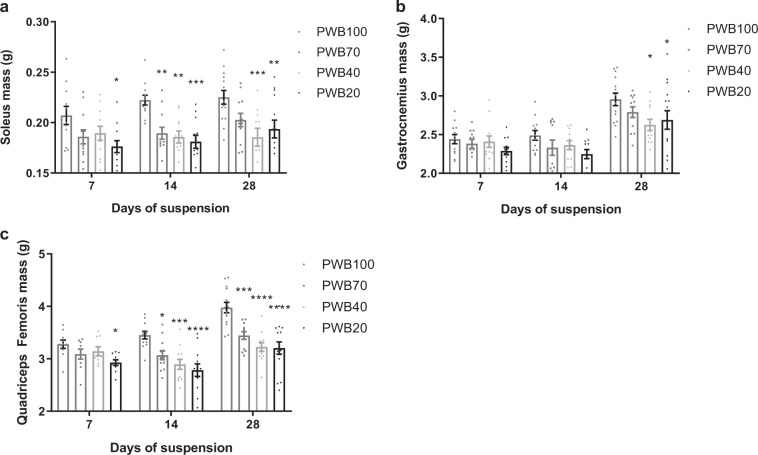
Muscle wet mass after 7, 14, and 28 days of PWB. Wet mass of several hind limb muscles after exposure to PWB, including soleus (**a**, *n* = 12–15 per group), gastrocnemius (**b**, *n* = 12–15 per group) and quadriceps femoris (**c**, *n* = 9–15 per group). Results are presented as mean ± SEM and Tukey’s post hoc tests following ANOVAs are represented as *: *p* < 0.05, **: *p* < 0.01, ***: *p* < 0.001 and ****: *p* < 0.0001 vs PWB100

At each time point (i.e., 7, 14, and 28 days) hind limb muscles were dissected and wet mass was recorded (Fig. 3). After 7 days, the PWB20 group showed a significant reduction in soleus mass compared to controls (−14.89 ± 2.87%, *p* < 0.05); PWB70 and PWB40 animals displayed substantial but non-significant reductions (−10.24 ± 3.20% and −8.54 ± 3.35%, respectively, Fig. 3a). After 14 days, all unloaded groups had a significantly lower soleus mass as compared to the PWB100 controls in a dose-dependent fashion. This finding remained observable at 28 days for the PWB20 and PWB40 groups but not for the PWB70 group despite a mean reduction of −9.53 ± 2.96% (*p* = 0.11, using a two-way ANOVA followed by Tukey’s post hoc test).

In contrast, the mass of the gastrocnemius muscle did not differ significantly for any PWB group as compared to PWB100 control animals for the first 2 weeks of the experiment (Fig. 3b). However, after 28 days of partial unloading, the animals exposed to PWB40 displayed significant muscle atrophy compared to the PWB100 controls (−11.14 ± 2.41%, *p* = 0.013, using a two-way ANOVA followed by Tukey’s post hoc test), as did the animals exposed to PWB20 (−9.00 ± 4.27%, *p* < 0.05). On day 7, quadriceps femoris wet mass (Fig. 3c) was significantly reduced only in the PWB20 group compared to the controls (−10.69 ± 1.66%, *p* < 0.05, using a two-way ANOVA followed by Tukey’s post hoc test). On days 14 and 28, all experimental groups displayed significant quadriceps atrophy compared to the controls at PWB100 in a clear dose-dependent fashion.

**Fig. 4:**
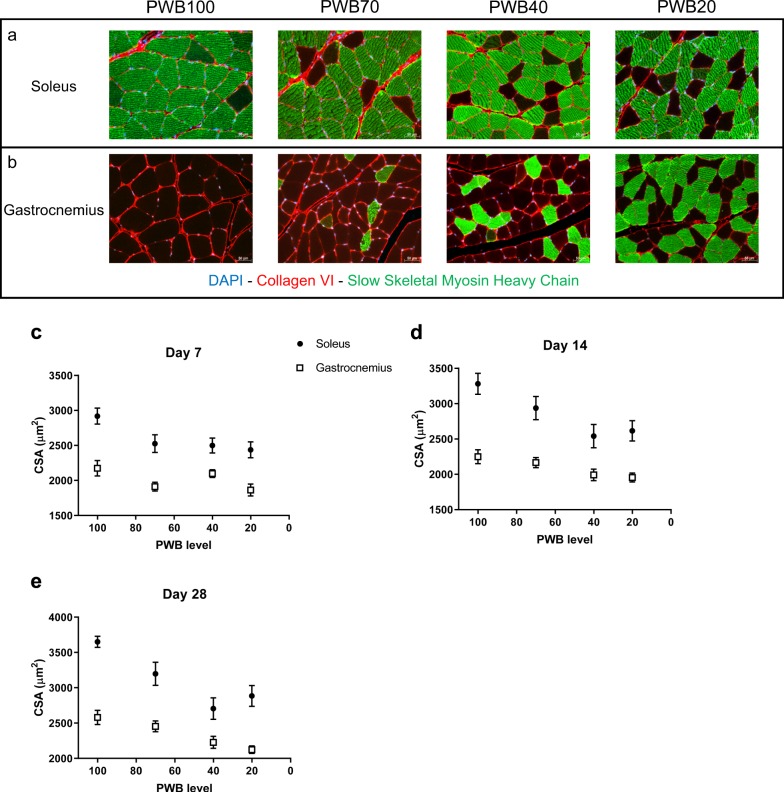
Histomorphometric analysis of the triceps surae. Immunofluorescence staining of the soleus (**a**) and the gastrocnemius (**b**) using antibodies targeting DAPI (blue), Collagen VI (red), and slow skeletal myosin heavy chain (green). Average cross-section area (CSA) of the soleus and gastrocnemius muscles after 7 days (**c**), 14 days (**d**), and 28 days (**e**) of PWB at all levels. Scale bars are indicated in the bottom right corner of each picture and represent 50 µm. Each muscle was analyzed across groups using a one-way ANOVA followed by a post hoc test for trend. Results are presented as mean ± SEM and post hoc tests for linear trend and *R*^2^ values of the fits are as follow. **a** Soleus *p* < 0.01, *R*^2^ = 0.79, Gastrocnemius *p* < 0.05, *R*^2^ = 0.39. **b** Soleus *p* < 0.01, *R*^2^ = 0.89, Gastrocnemius *p* < 0.01, *R*^2^ = 0.97. **c** Soleus *p* < 0.0001, *R*^2^ = 0.83, Gastrocnemius *p* < 0.0001, *R*^2^ = 0.99 (*n* = 11–12 per group)

Using quantitative histomorphometry, we measured the average cross-sectional area (CSA) of the myofibers in both soleus (Fig. 4a) and gastrocnemius (Fig. 4b) muscles, which have been extensively studied for their adaptation to microgravity. In both muscles and under all reduced PWB conditions and time points, i.e., 7 days (Fig. 4c), 14 days (Fig. 4d), and 28 days (Fig. 4e), the unloaded groups displayed a significantly smaller myofiber CSA than the PWB100 controls. Moreover, myofiber atrophy followed a significant linear trend across groups plotted against their degree of PWB (R^2^ values for the soleus are: 0.79, 0.89, and 0.83 for 7, 14, and 28 days, respectively. *R*^2^ values for the gastrocnemius are: 0.39, 0.97, and 0.99 for 7, 14, and 28 days, respectively).

Additionally, we observed a fiber type switch in these two muscles of the unloaded rats compared to the PWB100 controls (Supplementary Table 2). While accounting for only approximately 40% of the soleus myofibers in mice,^19^ type 1 fibers (i.e., slow-twitch, oxidative) normally comprise from 80 to 94% of the myofibers in the soleus muscle in humans and in rats^20,21^ We observed a similar percentage of type 1 myofibers in the soleus muscle in our control PWB100 group, ranging from 90.96 ± 1.25% to 88.68 ± 1.81% between the different time points. In contrast, the number of type 1 fibers decreased (and presumably the number of type 2 fibers (i.e., fast-twitch, glycolytic) increased) in the soleus muscle with decreasing PWB level. For example, after 14 days of suspension, the percentage of type 1 fibers decreased in the soleus to 85.32 ± 2.65% for the rats at PWB70, 78.51 ± 2.07% at PWB40, and 84.08 ± 2.13% at PWB20. The opposite trend was observed in the gastrocnemius muscle, which is composed predominantly of type 2 fibers in normal conditions (PWB100) and displayed an increasingly higher percentage of type 1 fibers in the increasingly unloaded groups. The fiber-type switch that we observed has been reported previously in the literature,^22,23^ and has been described as a preferential atrophy according to muscle location and fiber-type. The extent of fiber-type switching increases with time (*p* = 0.0019, using a two-way ANOVA followed by Tukey’s post hoc test) and reduction in PWB level (*p* = 0.0484, using a two-way ANOVA followed by Tukey’s post hoc test). However, although we visually observed a consistent trend in fiber-type switching in the gastrocnemius, we did not detect statistically significant differences between PWB groups in this muscle.

## Discussion

In this study, we demonstrate that our PWB model in rats shows excellent reliability and is effective in modeling the impact of reduced mechanical loading on muscle health, with several muscle parameters presenting dose-dependent alterations. The multiple time points allowed us to observe alterations in muscle function and mass as early as 7 days after exposure to reduced loading that appeared to become greater over time (Fig. 2b). The early muscle deficits that we observed here using our recently developed PWB model^18,24^ and are well described in the hindlimb unloading literature^25–27^ emphasize the need for mitigating strategies immediately after a reduction, even partial, in mechanical loading. Muscle impairment was further confirmed with the dose-dependent diminution in both the maximal torque obtained after a tetanic stimulation of the tibial nerve on day 28 (Fig. 2c), and the normalized tetanic impulse values at 28 days (Supplementary Table 1), therefore stressing the intrinsic loss of muscle function that correlated with reduced load. These physiological changes were also confirmed by the reduced hindlimb muscle mass (Fig. 3) and were associated with a reduction in myofiber cross sectional area (Fig. 4). These data are analogous to those previously observed after 21 days of PWB in mice.^15,16^ For example, when exposed to PWB20, soleus and gastrocnemius mass were reduced by 22% and 10%, respectively, compared to the control animals. Furthermore, similar to our findings, this previous work reported that the loss of muscle mass was dose-dependent and correlated to the PWB levels.

As in prior human and animal studies, both in space or using ground-based analogues,^4^ we observed that the soleus muscle atrophied to a much greater extent than the gastrocnemius with a significant decrease in type 1 slow-twitch oxidative fibers (Supplementary Table 2). Based on this immunostaining, we assumed that this was correlated to an increase in the proportion of fast-twitch glycolytic myofibers; however, this assumption should be confirmed in future work by specifically staining for fast-twitch fibers, which we did not do. This effect on muscle composition has been extensively studied in the context of microgravity^1,28,29^ although the changes do not always reach statistical significance.^30,31^ However, the non-significant trend we observed in the gastrocnemius (i.e., a visible increase in slow-twitch fibers as seen in Fig. 4b), which is a mixed-fiber type muscle, has only been described in the context of neuromuscular disorders such as spinal bulbar muscular atrophy.^32^ In this disorder, the cellular shift often precedes symptoms including muscle atrophy, and is thought to be caused by a metabolic dysfunction. Indeed, in this pathology, early changes in gene expression seem to be responsible for the alteration of the glycolysis process, which in return, is contributing to the myofiber type shift by modifying skeletal muscle metabolism and substrate availability. Analogously, prediabetic and metabolic symptoms such as insulin resistance are often experienced by astronauts during their missions.^33,34^ Thus, it would be interesting to further investigate the metabolic changes that are induced in our model, including skeletal muscle insulin-resistance, mitochondrial respiration and overall energy homeostasis, to determine if these mechanisms, which are known to impact the muscle tissue, contribute to the shift in myofiber type or are a consequence of it. Our future studies based on RNA sequencing, and aiming at unraveling the modulation of gene expression during PWB, will need to assess these additional parameters to better understand the underlying mechanisms.

Our PWB model offers the possibility of assessing the time-course of the musculoskeletal alterations across the spectrum of mechanical loading to better understand the underlying mechanisms of muscle loss and performance deficits. Muscle force, and therefore function, is a critical aspect of the astronaut health since it is essential for the efficient performance of a wide range of tasks. While reductions in force production may cause only minor issues during periods of microgravity, such deconditioning could represent a major impairment after landing, when astronauts are suddenly re-exposed to gravity. Interestingly, our results highlight that even a 30% decrease in mechanical loading (i.e., PWB70) induces substantial reductions in muscle force and size that do not recover after a month of suspension (Fig. 2c). The effects of partial gravity, as would be experienced by astronauts visiting Mars or the Moon or via centrifugation-induced artificial gravity, are far less studied than the effects of complete weightlessness due to the prior lack of established animal and human models. The ground-based PWB model we developed will allow for further investigation of the potential health impacts of partial mechanical unloading and could represent the first step toward finding solutions to protect astronauts during long-duration space missions.

While successful, there remain several limitations to this model. First, we observed an initial transient decline in body mass; and although within an acceptable range (i.e., <10% of the initial body mass), as it is experienced in all ground-based models as well as in astronauts themselves,^35–38^ this body mass loss can create some bias in non-invasive assessments such as limb girth. Second, unlike humans, rats display constant growth, which also involves the musculoskeletal system. Indeed, the increase in muscle wet mass and in the average myofiber cross-sectional area was visible in our cohort of fully-loaded (PWB100) controls between different time-points. However, it has been described that after the initial and rapid growth period, male rats grow at a constant rate between 10 and 30 weeks old before showing signs of aging,^39^ thus confirming our choice in using 14–18-week-old animals.

Another limitation of this work is that we did not attempt to quantify nocturnal or daytime behavior. However, in order to get some sense of their overall behavior, video monitoring was installed within the animal facility and qualitative differences in behavior among groups were not observed. Despite being quadrupedal animals, rats briefly but regularly display some bipedal behavior, mainly occurring during environmental exploration and feeding, and such bipedal behavior was visible in all groups. While not noticeably greater in the reduced PWB groups, these brief bipedal occurrences represent a change in the animals’ unloading status that was not preventable. Moreover, due to their quadrupedal locomotion, many factors diverge between humans and rats, and this model cannot account for all such differences. Additionally, we also did not obtain a continuous measure of ground reaction forces. This should certainly be considered for future work. Finally, we have not fully characterized the stress levels in our animals. While our behavioral assessments, including food intake, grooming, and physical assessments were helpful and are widely used to identify whether animals are under stress, it will be necessary to sample blood and/or feces at different time points to assess the level of corticosterone for an accurate measurement of the animals’ stress.

This model has a number of potential uses beyond simply providing a ground-based analogue for different levels of partial unloading. First, it can serve as a testing ground for studies of therapeutic agents or exercise countermeasures in partial unloading.^40^ This is especially true since, if needed, it is extremely easy to remove the animals from the PWB harness for short periods of time to administer medications by injection or perform other assessments. Similarly, for exercise countermeasures, the model allows for relatively normal positioning and thus it would be possible, for example, to outfit a cage with a treadmill apparatus, allowing animals to perform in normal or reduced loading conditions. Likewise, by incorporating other hazardous events that occur during space flight, such as radiation exposure, the model could also address the impact of the combined effects of PWB and radiation exposure to muscle health.^41,42^ Moreover, the model could be used as a tool to assess the impact of sequentially altering loading levels. For example, a fully hind-limb suspended rat could then be placed in PWB40, so as to mimic an astronaut traveling from Earth to Mars and then landing on Mars, assessing the effect of partial unloading on an already compromised musculoskeletal system.^24^ Alternatively, the model could be used to assess the impact of the gradual mechanical reloading, via the progressive reintroduction of increased PWB, as means of re-acclimating safely to full Earth gravity after an extended period in space.

In summary, we developed a PWB for rats capable of simulating various mechanical loading environments while maintaining a quadrupedal stance. This model, first developed to investigate the impact of reduced weight-bearing environments observed at extra-terrestrial targets such as the Moon or Mars, additionally brings valuable insights about the potential mitigating effects of applying artificial gravity during spaceflight to preserve muscle health.

## Methods

### Animals

Wistar male rats, *n* = 149, (Charles River Laboratories Inc., Wilmington, MA, USA) weighing a mean ± standard error of 408 ± 0.15 g (14 weeks of age) were obtained and housed in a temperature-controlled (22 ± range of 2 °C) room with a 12:12-h light–dark cycle. Water and rat chow were provided ad libitum and monitored daily. Rats were regularly assessed for any signs of pain or discomfort (e.g., porphyric staining around the eyes, poor grooming, and hair loss), harness and jacket fitting, ability to walk and move across the cage, food and water intake. Additionally, fully loaded and partially loaded mass measurements were recorded daily. All experimental protocols were approved by the Beth Israel Deaconess Medical Center Institutional Animal Care and Use Committee.

For all experiments requiring anesthesia, vaporized isoflurane (1.5–3.5%) + oxygen was used and body temperature was maintained using a water-blanket (Gaymar, Inc., Orchard Park, NY) set at 37 °C (Fisher Scientific, Hampton, NH, USA). At the end of the study, rats were euthanized by CO_2_ inhalation and hind limb muscles were harvested and weighed on a precision analytical balance (Fisher Scientific, Pittsburgh, PA).

### PWB suspension model

Animals were placed in four different groups: normal loading (PWB100, *n* = 38), 70% of normal loading (PWB70, *n* = 36), 40% of normal loading (PWB40, *n* = 36), and 20% of normal loading (PWB20, *n* = 39). In each group, 12 animals were euthanized on day 7 and day 14 for ex vivo tissue analysis. On day 28, 12–15 animals per group were euthanized. At the beginning of the study, rats were distributed to ensure a balanced distribution of the body weights across all groups. Forty-eight hours prior to day 0 (baseline) assessment, rats were placed in custom cages and jackets for acclimation. Beginning on day 0, and after obtaining the pre-suspension measurements, animals were subjected to either normal loading (PWB100) or other PWB levels using the model designed in our previous work.^18^ Briefly, rats were secured in a tether jacket and a pelvic harness and submitted to a 2-point suspension allowing them to maintain quadrupedal behavior while weighing 100, 70, 40, or 20% of their initial body weight. Animals were weighed daily and if necessary the PWB apparatus was adjusted to ensure that their achieved unloading level remained constant within a 5% margin error of their desired PWB level, by modulating the chain length of the suspension device. Rats were positioned over a digital animal scale (ZIEIS, Lakeville, MN, USA) and their mass at PWB100, including the mass of the suspension device, was recorded. Then, the “unloaded” mass was obtained and the recorded level of PWB was calculated every morning by using the ratio of these two measurements.

### Calf circumference

Under isoflurane anesthesia, animals were placed in a prone position with the left hind limb positioned at a 45° angle to the spine. The left leg was shaved using a hair clipper (Braintree Scientific, Braintree, MA, USA), and three measurements of the calf circumference were obtained at the tibial mid-shaft using a suture thread at baseline and after 7, 14, and 28 days of suspension. Each thread was then measured with a micrometer (Fisher Scientific, Hampton, NH, USA) and the average of the three measurements was recorded.

### Rear paw grip force

Rats rear paws were placed on a 50 N capacity digital grip force meter (Chatillon, Largo, FL, USA) and gently pulled backward until the animal released its grip. The peak force generated was measured by a force transducer linked to the bar. Three tests were performed with a short rest period of at least 30 s between each trial, and averaged. Results were expressed as the percentage of each animal’s grip force measured at baseline (i.e., pre-suspension).

### Foot plate plantar force generation

Under anesthesia, rats were placed on a force plate (Dual Mode Muscle Lever System, Aurora Scientific, Aurora, ON, Canada), and their left foot was securely taped on the foot plate. Monopolar electrodes (28 G, Natus Medical Incorporated, Pleasanton, CA, USA) were used to deliver a tetanic, supramaxial stimulation of the tibial nerve at the popliteal fossa at a frequency of 200 Hz, for 200 ms. The maximal torque response was recorded and the AUC was calculated.

### Muscle histomorphometry

After euthanasia, the left gastrocnemius, soleus, and quadriceps femoris muscles were harvested and the wet mass of each muscle was determined using a precision analytical balance. The soleus and gastrocnemius muscles were then placed in 10% buffered formalin and fixed for 48 h (histological analysis was not performed on quadriceps femoris). Samples were embedded in paraffin blocks, sectioned into 10 micron slices and double immunofluorescence staining was performed using anti-collagen VI antibody (ab6588, Abcam, Cambridge, MA, USA) and anti-slow skeletal myosin heavy chain antibody (ab11083, Abcam, Cambridge, MA, USA). Stained slides were subsequently imaged at 20× using a Zeiss Axio Imager M1 epifluorescence microscope and fiber area was measured using the muscle morphometry plug-in (Anthony Sinadinos using Eclipse IDE) and FIJI (FIJI, ImageJ, NIH) with examiners blinded to PWB level and duration.

### Statistical analysis

Data were analyzed with Graphpad Prism 7.2 (Graphpad Software, La Jolla, CA) using one-way ANOVA for terminal measurements, followed by Tukey’s post hoc test or the linear test for trend. Longitudinal data were analyzed using two-way ANOVA followed by Tukey’s post hoc test. Results are presented as mean ± SEM and considered significant when *p* < 0.05.

### Reporting summary

Further information on research design is available in the Nature Research Reporting Summary linked to this article.

## Supplementary information

Supplementary tables.

Reporting Summary

## Data Availability

The authors declare that data supporting the findings of this study are available within the paper and its supplementary information files.

## Supplementary information

**Supplementary information** accompanies the paper on the *npj Microgravity* website (10.1038/s41526-019-0080-5).
