# Supplementary material for: Longitudinal time course of muscle impairments during partial weight-bearing in rats
Source: NPJ Microgravity. 2019 Aug 22;5:20. doi: 10.1038/s41526-019-0080-5 (PMC6706399; doi:10.1038/s41526-019-0080-5)
Supplement: Supplementary file 1 — Supplementary tables. [file 41526_2019_80_MOESM1_ESM.pdf]

Supplementary Material

Supplementary Table 1: Normalized Tetanic Impulse at 28 days (mN.sec.g-[1])

Tetanic impulse recording at 28 days was normalized by the wet mass of the triceps surae for each animal to provide a value of the intrinsic force. Results are presented as mean ± SEM. n=3-6 per group.

**Groups**	**PWB100**	**PWB70**	**PWB40**	**PWB20**
	9.87 ± 1.64	7.68 ± 0.46	7.90 ± 0.59	6.17 ± 1.25

Results were analyzed using a 1-way ANOVA, followed by the post hoc test for linear trend (p=0.043)

Supplementary Table 2: Percentage of type 1 skeletal myofibers in the triceps surae muscles

Histomorphometry was used to quantify the number of type 1 slow-twitch skeletal fibers in the soleus and gastrocnemius of our animals and assess muscle composition. Results are presented as mean ± SEM, n=11-12 per group.

	**PWB100**	**PWB70**	**PWB40**	**PWB20**
**Day 7**				
Soleus	90.96 ± 1.25	88.28 ± 2.36	89.15 ± 1.85	90.19 ± 2.62
Gastrocnemius	10.73 ± 1.93	11.43 ± 1.93	12.52 ± 1.73	10.92 ± 1.76
**Day 14**				
Soleus	86.94 ± 2.41	85.32 ± 2.65	78.51 ± 2.07	84.08 ± 2.13
Gastrocnemius	10.1 ± 1.67	11.07 ± 1.07	10.18 ± 1.78	12.46 ± 1.81
**Day 28**				
Soleus	88.68 ± 1.81	85.85 ±2.36	82.39 ± 3.08	84.78 ± 3.46
Gastrocnemius	11.53 ± 1.58	12.18 ± 2.01	14.89 ± 2.88	12.86 ± 2.40

Results were analyzed using 2-way ordinary ANOVA followed by Tukey's post hoc test.
Gastrocnemius: ns. Soleus: Effect of time p=0.0019 Effect of PWB level p=0.048
